# Salt Reduction Initiatives around the World – A Systematic Review of Progress towards the Global Target

**DOI:** 10.1371/journal.pone.0130247

**Published:** 2015-07-22

**Authors:** Kathy Trieu, Bruce Neal, Corinna Hawkes, Elizabeth Dunford, Norm Campbell, Rodrigo Rodriguez-Fernandez, Branka Legetic, Lindsay McLaren, Amanda Barberio, Jacqui Webster

**Affiliations:** 1 Food Policy Division, The George Institute for Global Health, The University of Sydney, Sydney, NSW, Australia; 2 Policy and Public Affairs, World Cancer Research Fund, London, United Kingdom; 3 Department of Medicine, Libin Cardiovascular Institute, University of Calgary, Calgary, Alberta, Canada; 4 International SOS Freeport Industrial Public Health and Malaria Control, Kuala Kencana, Papua, Indonesia; 5 Unit of Noncommunicable Diseases and Disability, Department of Noncommunicable Diseases and Mental Health, Pan American Health Organization- WHO, Washington DC, United States of America; 6 Cumming School of Medicine, University of Calgary, Calgary, Alberta, Canada; University of Utah, UNITED STATES

## Abstract

**Objective:**

To quantify progress with the initiation of salt reduction strategies around the world in the context of the global target to reduce population salt intake by 30% by 2025.

**Methods:**

A systematic review of the published and grey literature was supplemented by questionnaires sent to country program leaders. Core characteristics of strategies were extracted and categorised according to a pre-defined framework.

**Results:**

A total of 75 countries now have a national salt reduction strategy, more than double the number reported in a similar review done in 2010. The majority of programs are multifaceted and include industry engagement to reformulate products (n = 61), establishment of sodium content targets for foods (39), consumer education (71), front-of-pack labelling schemes (31), taxation on high-salt foods (3) and interventions in public institutions (54). Legislative action related to salt reduction such as mandatory targets, front of pack labelling, food procurement policies and taxation have been implemented in 33 countries. 12 countries have reported reductions in population salt intake, 19 reduced salt content in foods and 6 improvements in consumer knowledge, attitudes or behaviours relating to salt.

**Conclusion:**

The large and increasing number of countries with salt reduction strategies in place is encouraging although activity remains limited in low- and middle-income regions. The absence of a consistent approach to implementation highlights uncertainty about the elements most important to success. Rigorous evaluation of ongoing programs and initiation of salt reduction programs, particularly in low- and middle- income countries, will be vital to achieving the targeted 30% reduction in salt intake.

## Introduction

Cardiovascular disease (CVD), the leading cause of death worldwide, kills 17 million people each year which represents 30% of all global deaths [1]. The major risk factor for cardiovascular disease is high blood pressure [1, 2], and excessive sodium intake is an important cause [3]. Sodium consumption of more than 2g/d is estimated to cause 1.65 million cardiovascular related deaths each year, representing around 1 of every 10 deaths from cardiovascular causes [4]. The World Health Organization (WHO) has recommended salt reduction as a ‘best buy’, recognising it as one of the most cost effective and feasible approaches to prevent non-communicable diseases (NCDs) [1].

A review of salt reduction strategies undertaken in 2010 identified 32 national salt reduction strategies worldwide [5]. Most were in Europe, followed by the Western Pacific Region and the Americas. Since the last review, a series of recent research reports have provided further support for salt reduction [3, 6–9] and while several widely critiqued observational analyses have contested the nature of the association between salt and vascular outcomes [10–13] the totality of the evidence supports efforts to achieve population-wide lowering of salt intake. Furthermore, the evaluation of the United Kingdom’s (UK) salt reduction strategy has highlighted feasibility, demonstrating a 15% reduction in population salt intake between 2003 and 2011 [14] with average blood pressure in the adult population falling by 3/1.4mm Hg over the same period [15].

In 2011, the United Nations (UN) General Assembly convened a High-Level Meeting to address the Prevention and Control of NCDs worldwide [16]. The General Assembly adopted the Political Declaration of the meeting, which committed all 193 member countries and states to the prevention and control of NCDs [17]. Subsequently, at the 66^th^ World Health Assembly, WHO Member States adopted the global target of a 30% reduction in mean population intake of salt/sodium by 2025. This was one of nine voluntary global targets set to achieve an overarching 25% reduction in premature mortality from CVDs, cancer, diabetes and chronic respiratory diseases by 2025 [18]. Subsequently the WHO has supported Member States by identifying how best to develop, implement and monitor salt reduction strategies [19, 20] including consideration of how salt reduction and iodine deficiency elimination programs can be integrated [21]. A range of resources have been made available [22, 23]. Within this context we sought to systematically document existing national salt reduction strategies and provide an overview of initiatives in place to reduce population salt intake in line with the new global target.

## Methods

### Search Strategy

Salt reduction initiatives were identified from a search of peer-reviewed and grey literature published up to May 2014 as well as from salt reduction experts and country program leaders.

The retrieval and summary process was divided into 5 steps:

1. Conduct a systematic search of peer-reviewed and grey literature
2. Establish a database to systematically record initiatives according to a pre-defined framework
3. Consult with international salt reduction experts
4. Supplement and verify information through questionnaires sent to country program leaders
5. Analyse and compare the findings with a similar review that was conducted in 2010.

We searched the following databases: Cochrane Central Register of Controlled Trials (CENTRAL), Cochrane Public Health Group Specialized Register, MEDLINE, EMBASE, Effective Public Health Practice Project Database, Web of Science, TRoPHI databases and LILACS database [24]. A comprehensive list of search terms (S1 Annex) was used including ‘salt’, ‘sodium’, ‘government programs’ or ‘nutrition policy’. Titles and abstracts were screened independently by two reviewers and inconsistencies (few) were resolved via discussion.

In parallel, a search for pertinent grey literature using these terms was conducted in OpenGrey, Google, WHO and regional office databases and websites, governmental websites (e.g., Food Standards Agency, Public Health Agency of Canada, Centers for Disease Control and Prevention), scientific or non-governmental organization (NGO) websites (e.g., Institute of Medicine, Food Safety Authority of Ireland, Heart Foundation) and international or national salt reduction associations (e.g., World Action on Salt and Health).

**Fig 1:**
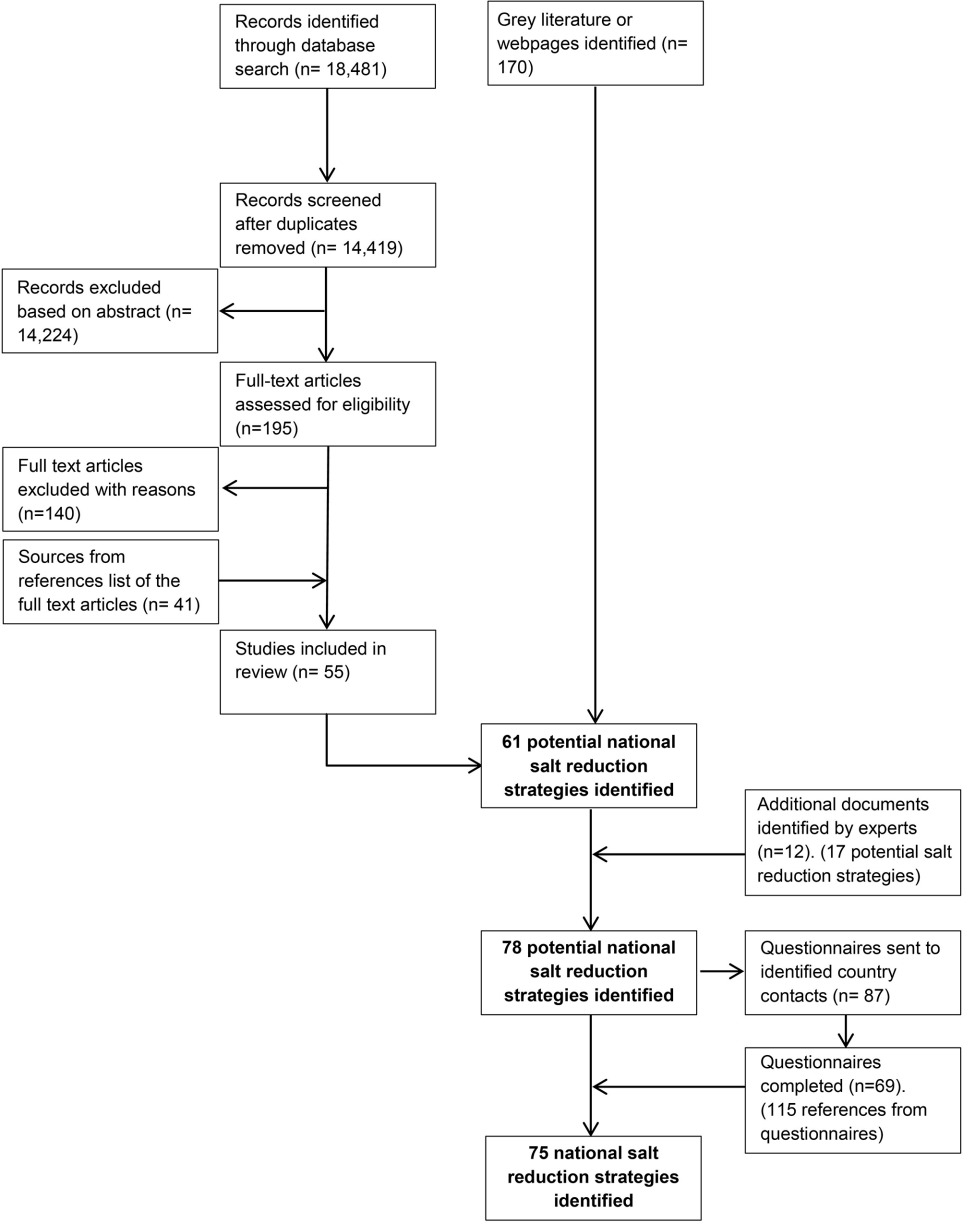
Identification process of national salt reduction strategies around the world.

A database of initiatives was established based on this information. A list of countries identified as having salt reduction strategies was sent to international experts and WHO representatives to identify whether any other countries with strategies had been missed. The database was continuously updated with the information being received. A questionnaire for each national country program was prepared (S2 Annex) by pre-filling each questionnaire based on existing country information. The questionnaire was sent to 86 country program leaders identified through the expert review. Additional information gathered from the responses was used to update the database. Queries were followed up with country program leaders, the relevant WHO regional expert or a targeted search (Fig 1).

### Ethics Statement

Program leaders were informed of the purpose of the study through an introductory email sent with the questionnaire and consented to the information being used as part of the study through return of the questionnaire. University of Sydney Human Research Ethics Committee approval was granted for the questionnaire portion of this work (#14923).

### Inclusion/Exclusion criteria

National strategies aiming to achieve population-wide reduction in salt intake were included. A national salt reduction strategy was defined as having government involvement with at least one of the following; a document and/or statement highlighting their commitment to population-level salt reduction OR a program of work to engage the industry to reduce salt in foods OR a campaign to change consumer knowledge, attitude & behaviour (KAB) specifically related to salt OR work aiming to reduce salt intake in public institution settings (e.g. government-funded schools, hospitals or workplaces). Initiatives that just provided information to consumers about salt as a part of a broader nutrition or health promotion campaign were excluded. National strategies were considered to be in their planning stages if initiatives were still being developed or if the strategic action plan had been developed but there was no evidence of program implementation.

### Data extraction

For each country, standard information relating to the characteristics of the national salt reduction strategy was extracted based on a previous review [5]. However, to improve the understanding of how national salt reduction strategies have progressed, additional information was extracted regarding targets for salt levels within specific food types, salt taxes and work in public institution settings. Information on reported program impact based on population-level salt intake, salt levels in foods and consumer KAB outcome measures was also gathered (S3 Annex).

### Analysis

Key characteristics for each national salt reduction strategy were entered in a database and examined in relation to: regional distribution as categorised by the WHO regions; income level classified by The World Bank; leadership and strategic approach; baseline monitoring data; types of implementation strategies; and evaluation of program impact. National strategies were classified as ‘strategy developed’, ‘strategy planned’ or ‘no strategy developed’ based on the strategies implemented and the country contact’s opinion. Where there were multiple sources of varying information, data was extracted from the source likely to be the most accurate or recent. A quantitative assessment of the percentage of countries reporting each characteristic was undertaken.

Where characteristics and information about the strategy were collected in both the current and previous 2010 review, there was a comparison to determine if there had been any changes.

## Results

### Sources of information

A total of 55 peer-reviewed articles and 170 grey literature documents and websites were retrieved from the literature search. An additional 115 documents were referenced in the completed questionnaires or obtained from a targeted search for more information to support questionnaire responses (Fig 1).

From the initial literature search, 61 countries with national salt reduction strategies were identified. International experts and WHO regional representatives provided 12 additional documents, which identified a further 17 potential national strategies. 87 questionnaires were sent to country program leaders and of these, 69 countries completed the questionnaire or provided relevant information. National salt reduction strategies were confirmed through 62 returned questionnaires and 7 confirmed there was no strategy or the strategy was still being planned. An additional 13 countries were considered to have a national salt reduction strategy based on information from peer-reviewed and grey literature despite not providing completed questionnaires.

### Countries with national salt reduction strategies by WHO region

**Fig 2:**
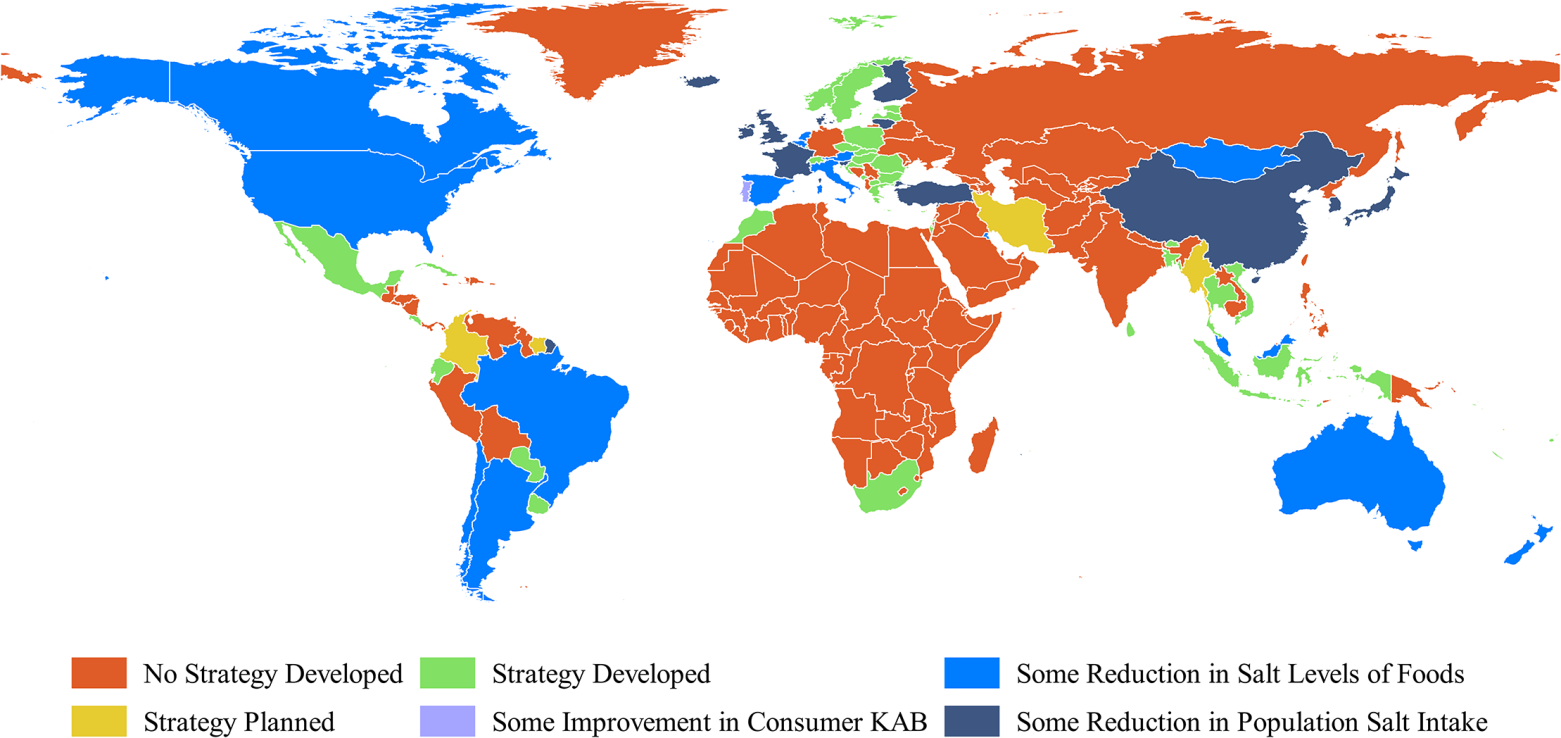
Countries’ reported progress towards reduction in population salt intake. Made with Natural Earth. Free vector and raster map data @ naturalearthdata.com.

In all, 75 countries (includes territories and areas) with national salt reduction strategies were identified in 2014, which is more than double the 32 reported in 2010. An additional nine countries are currently in their planning stages. National salt reduction strategies are now being implemented in countries across all six WHO regions and there are one or more strategies in the South-East Asia, Eastern Mediterranean and African region, where there were none previously (Fig 2).

**Fig 3:**
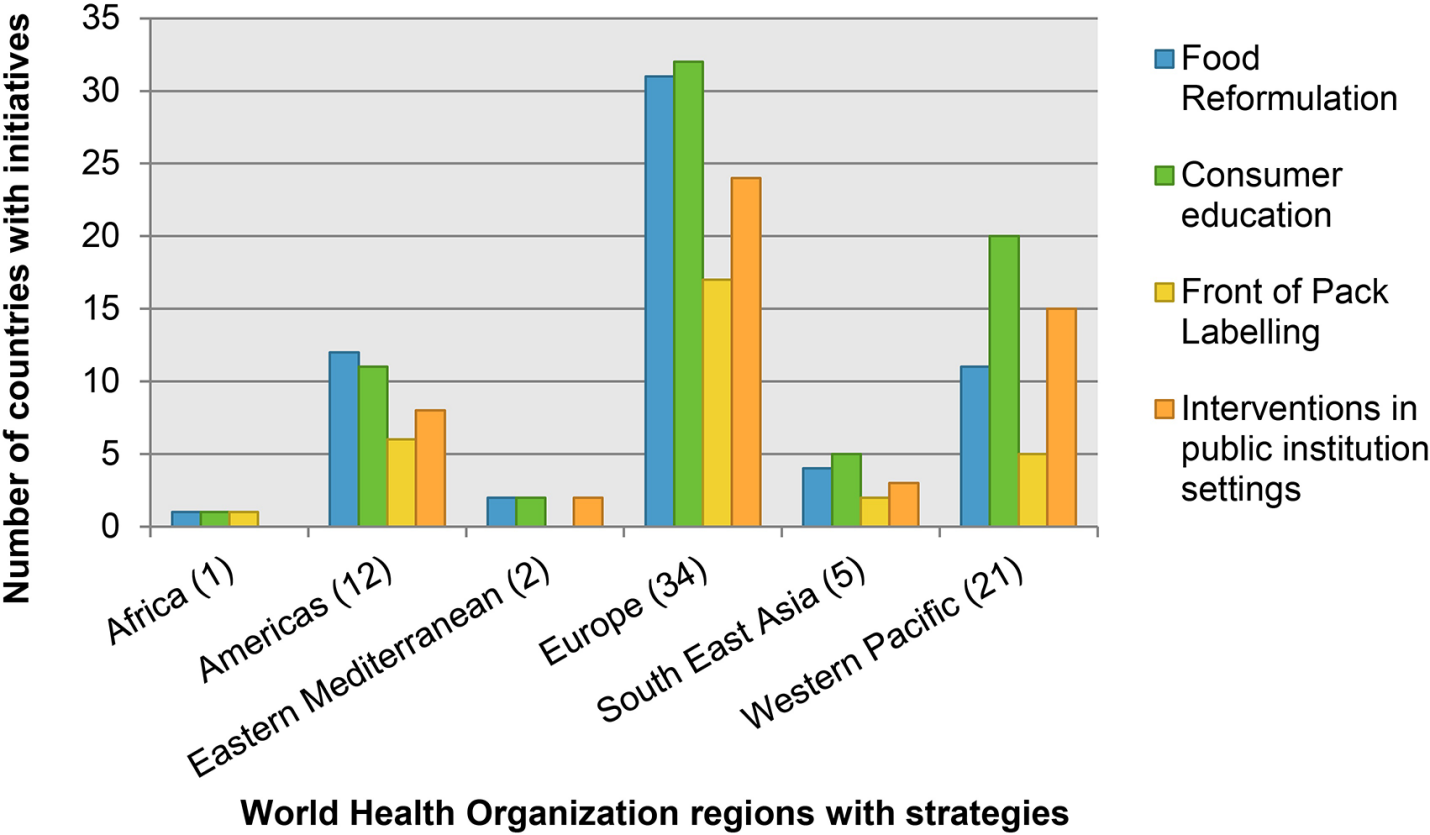
Types of salt reduction initiatives adopted by countries.

In all regions, consumer education was the most commonly used strategy followed by food industry engagement to reformulate products and FoPL schemes, except in the WHO Region of the Americas where industry engagement is the most commonly used strategy (Fig 3). The WHO South-East Asia Region is the only region where countries have not reported using voluntary or mandatory salt level targets to encourage reformulation (Fig 3).

### Countries with national salt reduction strategies by income level

National salt reduction strategies are now implemented in countries at all income levels as classified by the World Bank [25]. National salt reduction strategies identified previously [5] were limited to high income (25) and upper middle income countries (7). However strategies now exist in 41 high income countries, 21 upper middle income countries, 11 lower middle income countries, and 1 low income country (as well as 1 country that is not classified).

### Leadership and strategic approach

All strategies identified include some form of government involvement. However, 45 countries also report NGO, industry or other advocacy organization activity of which 9 acknowledged NGO or industry leadership. Three-quarters (57/75) of strategies have an established target for population salt intake, ranging from 5 to 8 grams per day. 38 countries have a target of 5 grams of salt per day, as recommended by WHO, 16 countries have a target of 6 grams per day and 3 have targets of more than 6 grams per day. The majority (72%) of national salt reduction strategies are linked to a broader initiative such as non-communicable disease or nutrition programs. There have been no significant changes in the leadership and strategic approach of national salt reduction strategies since the previous review in 2010.

### Baseline assessment and monitoring

With regards to establishing a baseline, 60 countries have one or more estimates of population-level salt intake, approximately double the number of countries compared to 2010. 24-hour urine collection, regarded as the gold standard for assessing salt intake, has been carried out in 32 countries, while 42 have used dietary surveys including 24-hour dietary recall or food frequency questionnaires. 7 have used household budget surveys to estimate salt intake. Whilst dietary surveys are still the most common method of estimating population salt intake, there has been an increase from 7/32 (22%) to 32/75 (43%) in the use of 24-hour urine samples since the previous review. The most current reported salt intakes ranged from 5 grams in Cyprus measured by dietary survey in 2005–08 [26] to 15.3 grams in a province of Vietnam, measured by spot urine in 2012. Out of the 60 countries with data available, 27 reported mean salt intake levels equal to or greater than 10 grams (double the WHO recommended daily salt intake).

Data regarding the sodium content level of some foods or meals has been collected in 52 countries, an increase from 56% to 69% since the 2010 review. Sodium content data is collected through food composition databases, food analysis, industry self-reporting or shop surveys.

Of the 75 strategies identified, the proportion measuring KAB in relation to salt at baseline, increased substantially to 60% (45/75), compared to 13% (4/32) in the 2010 review. Most surveys included questions relating to knowledge of the adverse health effects of excessive salt intake, attitudes towards the importance of reducing salt or behaviour such as adding salt to food without tasting or cooking with low-salt products.

### Implementation strategies

The main implementation strategies for salt reduction are food reformulation, consumer education, front of pack labelling, interventions in public institution settings (such as schools, hospitals and the workplace) and taxation (Table 1). Almost all countries are multifaceted in their approach, with 70/75 (93%) countries implementing or planning to implement more than one type of strategy. Additionally more countries are incorporating legislative initiatives (33/75) such as establishing maximum sodium content limits in foods, taxing high-sodium content products, mandatory FoPL schemes or warning labels for high salt foods and sodium content standards for publicly procured foods and meals (Table 2). So far, only two countries, South Africa and Argentina, have adopted comprehensive legislative schemes to limit salt levels in foods [27, 28].

**Table 1:** Baseline assessments and implementation strategies by WHO Region.

Country	Monitoring		Implementation Strategies			
	Baseline assessment	Salt intake (g/person/day)	Food reformulation	Consumer education	Front of Pack Labelling	Interventions in public institution settings
**World Health Organization African Region**						
Mauritius (P) [47]	SI/SL	7.9 (2012 24h)	P	P	P	E/VG (sch/wk/ hosp)
South Africa [27, 48, 49]	SI/SL/KAB	8.1 (2005 24h & DS)	T (M)	NGO	Logo (vol)	P
**World Health Organization Region of the Americas[50, 51]**						
Argentina [52]	SI/SL/KAB	11.2 (2011 24h)	T (vol/M)	Gov	No	E/PP/VG (sch/wk)
Barbados [20]	SI/SL/KAB	12–15 (2010 unknown)	IM	Gov/ NGO	No	E/VG (sch/wk)
Brazil [53]	SI/SL/KAB	11.8 (2002–3 & 2008–9 HS)	T (vol)	Gov	No	E/PP/VG (sch)
Canada [54]	SI/SL/KAB	8.5 (2004 DS)^a^	T (vol)	Gov/ NGO	Logo (vol)	No
Chile [55]	SI/SL/KAB	9.8 (2009–10 spot)	T (vol)	Gov	W (man)	E (sch)
Colombia (P) [56, 57]	SI^b^ ^#^	11.8 (1988 24h)^b^	P	P	No	-
Costa Rica [58]	SI/SL/KAB	9.3 (2004–5 HS)	IM	Gov	%DI (vol)	PP (sch)
Cuba	KAB	-	IM	Gov	-	PP (sch)
Ecuador [59]	SI/SL/KAB	10 (DS)	T (vol)	-	TL (man)	-
Mexico	No	P	T (vol)	Gov	%DI (man)	PP (sch)
Paraguay [60]	SL/KAB	P	T (M)	Gov	No	E (sch/wk)
Suriname (P) [61]	No	P	-	Gov	P	-
United States [62–64]	SI/SL/KAB	8.8 (2009–10 DS)	T (vol)	Gov/ NGO	% DI (vol)	E/PP/VG (sch/wk/ hosp)
Uruguay	SI/SL/KAB	9.5 (2005–6 HS)	T (vol)	Gov	P	No
**World Health Organization Eastern Mediterranean Region**						
Iran (P) [65]	SI^b^	10.6 (2007 24h)^b^	P	Gov	No	E (sch)
Kuwait	SI/SL	8.8 (m) 6.9 (w) (2010 DS)	T (Vol)	Gov	P	E/PP (sch/ wk/ hosp)
Morocco	SI/SL/KAB	Yes TBA (2013–14 24h & DS)	IM	NGO	P	No
**World Health Organization European Region [26, 29]**						
Austria [66]	SI/SL/KAB	8.7 (m) 7.6 (w) (2012 24h & DS)	T (vol)	Gov	No	VG (sch)
Belgium [67]	SI/SL/KAB	10.45 (2009 24h)	T (vol/M)	Gov	Logo (vol)	No
Bulgaria	SI/SL/KAB	13.8 (2004 DS)	T (vol/M)	Gov	%DI (vol)	PP (sch)
Croatia [68]	SI/SL	12 (2010 DS)	T(vol)(bread)	Gov/ NGO	No	VG (sch)
Cyprus	SI	5 (2005–8 DS)	IM	Gov	Yes (vol)	E (sch)
Czech Republic	SI/SL	13.6 (2003–4 DS)	T (vol)	Gov	Logo (vol)	-
Denmark	SI/SL	9.88 (m) 7.02 (w) (2010 median spot)	T (vol)	Gov	Logo (vol)	-
Estonia	SI/KAB	10 (1997 DS)	P IM	Gov	%DI (vol)	PP (sch/ hosp)
Finland [69, 70]	SI/SL/KAB	9 (m) 6.5 (w) (2012 DS)	T (vol)	Gov/ NGO	W (man) Logo (vol)	E/PP/VG (sch/ wk/hosp)
France [33]	SI/SL/KAB	8.4 (2006–7 DS)	T (vol)	Gov	No	PP (sch)
Greece	SL/KAB	No	T (vol/M)	Gov	No	E/PP (sch)
Hungary [71]	SI/SL/KAB	11.2 (m) 9.6 (w) (2010 24h)	T (vol/M) (bread)	Gov	Logo (vol)	PP (sch)
Iceland	SI	9.5 (m) 6.5 (w) (2010–11 DS)	IM (bread)	Gov	-	-
Ireland [72]	SI/SL/KAB	11.1 (m) 8.5 (w) (2008–10 DS & spot)^a^	T (vol)	Gov	%DI (vol)	E (sch)
Israel	SI/SL/KAB	7 (1999–2001 DS)	T (vol)	Gov	P	E/PP (sch/ wk/ hosp)
Italy [73]	SI/SL/KAB	10.6 (m) 8.2 (w) (2009–11 24h)	T (vol)	Gov/ NGO	No	P
Latvia [74]	SI/SL/KAB	7.1 (2007 DS)	IM	Gov	%DI (vol)	PP (sch/ hosp)
Lithuania [75]	SI/SL/KAB	8.75 (2007 DS)	T (vol)	Gov/ NGO	Logo (vol)	E/PP (sch)
Luxembourg	SI/SL	9.1 (2007–8 DS)	IM	Gov	-	E (sch)
Macedonia	SI	14 (2011 IE)	No	P	No	P
Malta [57]	SI^b^/KAB	9.7 (1988 24h)^b^	P IM	Gov	No	P
Montenegro	No	P	IM	-	-	-
Netherlands	SI/SL/KAB	10.7 (m) 7.8 (w) (2010 24h)	T (vol)/ T(M) (bread)	NGO/ Industry	Logo (vol) / %DI (vol)	VG (sch/ wk)
Norway	SI/SL/KAB	10 (2010–11 DS)	IM	Gov	Logo (vol)	P
Poland	SI/SL/KAB	10.9 (2009 IE)	T (vol)	Gov/ NGO	Logo (vol)/ GDA (vol)	E/VG (sch/ hosp)
Portugal [76]	SI/KAB/SL (bread)	10.7 (2012 24h)	T (vol/M) (bread)	Gov	TL (M)	E/VG (sch/ wk)
Romania	SI	11.25 (2010 DS)	IM	Gov	-	PP (sch)
Slovakia	SI	9.5 (m) 6.5 (w) (2011 DS)	IM	NGO	No	-
Slovenia [77]	SI/SL/KAB	11.3 (2012 24h)	T (vol)	Gov	-	E/PP (sch/ wk/ hosp)
Spain [78]	SI/SL	9.7 (2009 24h)	T (vol)	Gov	No	PP (sch)
Sweden	SI/SL	10–12 (2011 DS)	T (vol)	Gov	Logo (vol)	PP/VG (sch/ wk)
Switzerland [79]	SI/SL	9.1 (2011 24h)	IM	Gov/ NGO	No	VG (sch/ wk/ hosp)
Turkey [31]	SI/SL	15 (2012 24h)	T (vol)	Gov	Logo (P)	E/VG (sch/ wk/ hosp)
United Kingdom [80–82]	SI/SL/KAB	8.1 (2011 24h)	T (vol)	Gov	TL (vol) /%DI (vol)	E/PP (sch)
**World Health Organization South East Asia Region [83, 84]**						
Bangladesh	SI^b^/KAB	10–11 (2012 24h)^b^	IM (NGO)	NGO	No	No
Bhutan (short term strategies)	No	No	No	Gov	No	E (sch)
Indonesia (P)	SI	15 (2012 unknown)	IM	Gov/ NGO	%DI (vol)/ W (man)	P
Myanmar (Burma) (P)	SI	6–8 (2012 unknown)	No	Gov	No	-
Sri Lanka	SI^b^ /SL	8.3; 8.9 (urban; rural 2012 24h)^b^	IM/ P T (M)	Gov	TL (P)	VG (wk/ hosp)
Thailand [85]	SI/SL	10.8 (2009 DS)	IM	Gov	%DI (man)	E (sch/ hosp)
**World Health Organization Western Pacific Region [86]**						
Australia [87, 88]	SI/SL/KAB	8.9 (2011 24h)^b^	T (vol)	NGO	%DI/Logo (vol)	VG (sch/ wk/ hosp)
China [44]	SI/SL/KAB	12 (2009 DS)	No	Gov	%DI (vol)	-
Confederation of Northern Mariana Islands (P)	No	No	No	Gov/ NGO	No	E/PP/VG (sch)
Cook Islands	SI/SL/KAB	Yes (2013–14 24h)	T (vol)	Gov	No	E/PP (sch/wk)
FSM	No	No	IM	Gov	No	E (sch/ wk/ hosp)
Fiji	SI/KAB	9.4 (2012–13 24h)	T (vol)	Gov	P	E/VG (sch/ wk/ hosp)
French Polynesia	No	No	P	Gov	No	E (sch/ wk)
Guam (P)	No	P	P	Gov	P	P
Japan [43]	SI	10.4 (2012 DS)	IM	Gov/ NGO	No	No
Kiribati (P)	SL	No	No	Gov	No	No
Korea [89]	SI/SL/KAB	11.6 (2012 DS)	T (vol)	Gov	%DI (vol)/ TL (man)	E/PP (sch/wk)
Malaysia	SI/SL	6.4 (2003 DS)	IM	Gov/ NGO	P	E/PP (sch/ hosp)
Marshall Islands	No	No	No	Gov	P	E/VG (sch)
Mongolia [90]	SI/SL/KAB	11 (2011 24h)	T (vol)	Gov	-	E/VG (wk)
Nauru	No	No	No	Gov	No	E (sch)
New Caledonia	KAB	No	IM	Gov	No	E (sch/ hosp)
New Zealand [91]	SI/SL/KAB	8.57 (2012 24h)	T(vol) (NGO)	Gov/ NGO	%DI/Logo (vol)	No
Palau	No	P	No	P	No	P
Samoa (P)	SI/SL/KAB	6.9 (2013 24h)	P	Gov	P	P
Singapore [83, 92]	SI/SL/KAB	8.3 (2010 24h)	IM	Gov/ NGO	Logo (vol)	E/VG (sch)
Solomon Islands	No	No	P	Gov	No	P
Tonga	No	No	P	Gov	No	E (sch)
Tuvalu	SL	P	No	Gov	No	E (sch)
Vanuatu (P)	SL	No	P	P	No	E (sch)
Vietnam	SI^b^/SL/KAB	15.3 (2012 spot)^b^	P	Gov	No	E (sch)

P–Planned; SI-population salt intakes; SL–salt level in foods; DS–dietary survey; HS–household survey; 24h–24 hour urines; spot—spot urines; IE–indirect estimate of salt intake; KAB—consumer knowledge, attitude or behaviour; m–men; w—women

^a^ includes discretionary salt

^b^ not nationally representative

T—sodium content targets for foods; IM—industry meetings; Vol—voluntary; M–mandatory; NGO—non-governmental organization; Gov–government; %DI—percentage daily intake labelling (or Guideline daily amount in some countries); TL—traffic light labelling; W—high salt warning labels; E—education; PP—food procurement policy with sodium standards; VG—voluntary guidelines for sodium in foods; Sch—school settings; Wk—workplace settings; Hosp—hospital settings.

Dashes (-) indicate not aware of program. Each country’s population salt intake listed is based on the most current nationally representative assessment where available.

**Table 2:** Countries with legislative action on salt reduction.

**Mandatory Salt Targets**	Argentina (most foods) [28], Belgium (bread) [29], Bulgaria (bread, milk products, meat products & lutenica) [29], Greece (bread, tomato products) [29], Hungary (bread) [29], Netherlands (bread) [29], Paraguay (bread) [60], Portugal (bread) [29], South Africa (most foods) [27]
**Taxation on high salt foods**	Fiji (tax on MSG), Hungary [29], Portugal [29]
**Regulation on Front of Pack Labelling**	Chile [51], Ecuador [51], Finland [29], Indonesia [93], Korea (on children’s foods) [94], Mexico [51], Portugal [76],Thailand (on 5 snack food categories) [94]
**Standards for salt as part of procurement policies in public institution settings**	Argentina, Brazil, Bulgaria [95], Cook Islands, Costa Rica [58], Estonia [95], Finland, France, Greece, Hungary, Israel, Korea, Kuwait, Latvia [95], Lithuania, Malaysia, Mexico, Romania, Slovenia, Spain [96], Sweden, USA [97], UK [98]

#### Food Reformulation

Like the previous review, a high majority (81%) of national salt reduction strategies include industry engagement to reduce the salt content of products. Globally, bread is the most targeted food for reformulation followed by foods such as bakery products, processed meats, dairy products, sauces and convenience meals. 36 countries have taken the next step to establish voluntary sodium content targets for foods and meals. Furthermore, nine countries have mandated maximum sodium content limits for products. Whilst these are mostly for bread, Argentina, Bulgaria, Greece and South Africa have additional limits for other foods.

#### Consumer education

Raising consumer awareness and education in relation to salt is still part of most strategies. All except four countries have consumer education campaigns, 50 of which are led solely by government, 16 by both government and NGO or industry and 5 led solely by NGOs. In almost all cases, consumer awareness and education activities are used in conjunction with other salt reduction intervention strategies.

#### Front of Pack Labelling

A total of 31 countries (compared to 10 in 2010) have voluntary or mandatory FoPL schemes related to salt or sodium. Of those, eight countries have mandatory FoPL schemes. The most frequently used FoPL scheme are logos and symbols (19), which also includes traffic light labels (4), to indicate that the product meets established nutrient criteria. The second most commonly used FoPL scheme is the percentage daily intake (%DI) or guideline daily amount (%GDA), adopted by 16 countries. Three countries have warning labels on high salt foods. FoPL schemes have been introduced by government, NGO and industry.

#### Interventions in public institution settings

National salt reduction strategies involving activities that target public institution settings such as schools, workplaces, public hospitals and other public institutions were identified in 43 countries. This information was not previously reported in 2010 and does not take into account the fact that in some countries, state or local governments would have jurisdiction over this. Many of these activities are education programs; however a high proportion of countries (37/43) have nutrition guidelines for foods and meals sold and served in public settings, particularly schools and hospitals. 19 countries have voluntary nutrition guidelines and 23 have mandatory nutrition standards (including sodium) for foods and meals procured in public settings.

Taxation. Three countries; Fiji, Hungary and Portugal have adopted a tax related to salt. Two have a specific sodium tax and the third has a general tax. Fiji has a tax on monosodium glutamate (MSG) which it increased from 5% to 32% in 2012 [29]. Similarly, Hungary introduced a public health product tax on an extensive range of pre-packaged foods with high salt and sugar contents in 2011. The tax applies to products such as salty snacks with salt content above 1 gram per 100 grams, condiments with more than 5 grams per 100 grams and flavourings above 15 grams per 100 grams [29]. Portugal has a valued-added tax (VAT) on processed or packaged foods in general which covers foods high in salt, compared to a reduced VAT for non-processed foods [29].

### Reported Program impact

#### Change in population salt intake

12 countries have reported a reduction in population salt intake (Table 3). This is an increase from four countries identified in 2010. Moreover, three of the four countries (Finland, Japan and UK) that reported reductions in 2010 are reporting further reductions [14, 30]. Countries reported using different methods to monitor changes in sodium intake which may affect the strength of the evidence for reductions. Slovenia, Turkey and the UK reported salt intake reductions of 9%, 16% and 15% respectively, based on 24 hour urinary excretion measurements [14, 31]. Finland monitored population salt intake through both 24 hour urine collection (FINRISK) and dietary surveys (FINDIET surveys) [30]. Denmark monitored population salt intake from spot urine sodium to predict 24 hour sodium excretion [32]. The remaining countries used comparative dietary surveys to estimate change in salt intake. Reductions ranged from approximately 5% in France between 1999 and 2007 to 36% in Finland between 1979 and 2007 [30, 33]. For some countries such as China, Turkey and Lithuania, the baseline and follow-up data points do not align closely with the salt reduction implementation dates and therefore it is difficult to conclude that reductions were due to the interventions.

**Table 3:** Countries that have reported a reduction in population salt intake.

Country	Measurement tool	Reduction in population salt intake	Timescale
China[45]	Dietary survey	28.8% (16.8g to 12g)	1991–2009
Denmark	Spot urine	7% (10.68g(m),7.51g(w) to 9.88g(m), 7.02g(w)) (Median salt intake)	2006–2010
Finland[30]	Mixed (dietary survey & 24hr urine)	36% (13g(m),11g(w) to 8.3g(m),7g(w))	1979–2007
France[33]	Dietary survey	4.9% (8.1g to 7.7g)	1999–2007
Iceland[26]	Dietary survey	6.0% (8.4g to 7.9g)	2002–2010
Ireland[99]	Dietary survey	13.6% (8.1g to 7g)	2001–2011
Japan	Dietary survey	23.0% (13.5g to 10.4g)	1997–2012
Korea	Dietary survey	13.6% (13.37g to 11.55g)	2005–2012
Lithuania [75]	Dietary survey	18.6% (10.75g to 8.75g)	1997–2007
Slovenia	24hr urine	8.9% (12.4g to 11.3g)	2007–2012
Turkey [31]	24hr urine	16.7% (18.01g to 15g)	2008–2012
UK [14]	24hr urine	14.7% (9.5g to 8.1g)	2001–2011

#### Change in sodium content of foods

19 countries reported a change in salt levels in foods and meals in 2014 compared to 4 in 2010. Brazil and Kuwait have recently reported reductions in addition to the 17 countries previously recorded [34]. Changes in sodium content were measured by laboratory analyses in 12 countries, product label survey in two, industry self-report in three and both industry self-report and label survey in two countries. All countries except Malaysia reported that sodium content had declined in bread, with reductions ranging from 6% to 38%. 11 countries also reported that sodium content had declined in other foods such as processed meats, cheese, breakfast cereals, sauces, convenience and ready meals.

#### Change in consumer knowledge, attitudes and behaviour in relation to salt

Improvement in consumer KAB in relation to salt was reported in seven countries compared to two in 2010 (Table 4). All countries measured changes based on surveys either self- completed or in an interview.

**Table 4:** Examples of changes in consumer knowledge, attitudes and behaviour in relation to salt.

Country	Method of assessment	Improvements in consumer KAB
Ireland[100]	Consumer Survey	Behaviour- 37% of adults claimed they had changed behaviour as a result of the Salt Heart Campaign. 25% of adults claimed they changed their behaviour as a result of the ‘Already salted campaign'.
Korea	National Health and Nutrition Survey	Attitude- reported an increased interest in sodium in consecutive years based on the NHNS
Netherlands	Consumer Survey	Attitude- an increased number of people reported paying attention to the salt content of foods between 2008 and 2011
Portugal[101]	Survey	Knowledge & behaviour- the number of people who were aware of the risks of excessive salt intake increased from 29% in 2007 to 72% in 2009
Singapore[102]	Survey	Behaviour- the number of people NOT adding salt or sauce to food at the table increased from 39.8% in 1998 to 72.4% in 2010.
Slovenia	Computer Assisted Telephone Interviewing	Knowledge- the proportion of respondents who believe their daily consumption of salt is too high raised between April 2010 and February 2011
United Kingdom[81]	Survey	Behaviour- the number of adults claiming to make a special effort to reduce salt increased from 34% in 2004 to 43% in 2009

## Discussion

The number of countries with national salt reduction strategies has more than doubled since the previous review in 2010 with programs now implemented in countries across all WHO regions and across countries with a broad range of income levels. Whilst some of the additional 44 countries identified by this review may have commenced planning and activities prior to 2010, the majority (33/44) report start dates from 2010 onwards. This suggests significant impact has accrued through the recent efforts to expand the programs, driven by the World Health Organisation.

However, whilst the range of countries with programs in place has increased there remains marked variation in penetration across WHO regions with almost two-thirds of the European Region, but only 10% and 2% of the Eastern Mediterranean and African Region active. The coordinated actions of regional agencies such as the European Salt Action Network (ESAN) and the Pan American Health Organization (PAHO) were a likely key factor in the 80% increase and doubling of strategies in the European and Americas regions, respectively. Likewise the tripling of activity in the WHO Western Pacific Region reflects concerted WHO efforts. Additional support is now most urgently required in the Eastern Mediterranean and African regions where country engagement remains very low.

At present 33 low- and middle-income countries have a national salt reduction strategy in place, an increase from 7 in the previous report. The promotion of salt reduction as a ‘best buy’—an intervention that is not only cost-effective but also affordable, feasible and culturally acceptable to implement in any resource setting [1]–makes it a particularly compelling proposal for lower income settings. There is also potentially enormous impact of salt reduction in countries where there is a high salt intake combined with a very large population such as China and India.

### Increased legislative initiatives

There has been a shift from solely voluntary initiatives to incorporating a legislative or fiscal component (Table 2). In 2010, very few countries had mandatory levels for salt and only in a limited number of food products. South Africa and Argentina have led with the development of much more extensive schemes [27, 28]. Three salt-related taxes and six (of eight) mandatory FoPL schemes that include labelling of salt have also been recently implemented. Direct evaluation of the efficacy of these schemes is awaited but modelling studies consistently suggest that legislative action will be much more effective [35]. Robust monitoring and evaluation plans built into these programs from the start will be key to determining their utility and making the case for their more widespread use [34, 36].

Many countries are now reporting food procurement policies which incorporate salt standards for foods in public institution settings such as public schools and hospitals, as part of their national salt reduction strategies. A separate review of healthy food procurement policies has demonstrated positive impact in relation to availability and selection of healthy food [37] but further research is required to understand how such policies can be best used to lower population salt intake. This should include examination of the extent to which they can be used, not only to improve the nutritional composition of foods served but also in terms of the potential to generate an increased demand for healthier (low-sodium) foods.

### Approaches to monitoring

Implementing clear monitoring approaches is vital to demonstrate program effectiveness, but it also encourages greater change, particularly for voluntary strategies [34]. More countries have established mechanisms for monitoring of population salt intake levels, sodium content in foods and salt-related KAB but the quality of approaches vary.

Comparative dietary surveys were used in over half of the countries that reported reductions in population salt intake. Whilst this approach is likely to underestimate salt intake, if the method of measurement is consistent, it is still a useful measure of change over time [38, 39]. Additionally it can provide information about sources of sodium in the diet. Although 24 hour urine sodium excretion is considered the gold standard, it can be costly and a high burden on participants, which is challenging for many countries to adopt [20]. The use of spot urine measurements to estimate 24 hour urinary sodium excretion may therefore be more appropriate for large population surveys [39] and WHO has produced guidance for monitoring salt intake as part of STEPS that countries should follow [20]. This includes undertaking spot urine samples for the whole sample and 24 hour urine samples for a sub-sample of the population where possible, as well as standard questions for assessing and monitoring changes in KAB [40].

Standardised comparable approaches to measuring salt levels in foods are also vital. Almost two-thirds of countries monitoring change in salt levels in one or more food categories are using laboratory analysis which is likely to be very accurate but often covers a limited range of products rather than reflecting the whole food supply. Comprehensive surveys of salt levels in foods based on reported product label data should be considered in parallel to ensure that progress is made across a larger scale [34].

### Countries reporting an impact

Whilst 29 countries have reported an impact in relation to one or more of the outcome measurements, the different monitoring approaches means that the strength of the evidence varies. All but one of the programs reporting an impact to date are voluntary, however this is likely due to the fact that most legislative approaches to salt reduction are relatively new and there has been insufficient time to measure an impact. The exception is Finland, where the 36% reduction in population salt intake is partially attributed to its mandatory warning labels on high salt foods established in 1993, which led to significant reformulation of foods [26, 41].

Engagement with food industry including the establishment of specific salt targets for a range of foods is a key element of 10 out of the 12 countries that have reported a reduction in population salt intake.

The two countries which did not prioritise engagement with the food industry were Japan and China, where processed foods are not the major source of sodium in the diet. In Japan, most of the sodium in the diet comes from soy sauce added during cooking or eating, followed by salted vegetables and miso soup [42]. Therefore salt reduction efforts have been focused on improving people’s understanding of salt and health through public education and consumer awareness campaigns [43]. Likewise in China, the main approach has been through national and regional educational campaigns [44]. However, it is unlikely that these educational programs are the major cause of reduction in salt intake in China. Changes in the diet and food environment associated with modernization during this period, such as refrigeration and advancements in transport has led to a reduction in preservation and pickled food consumption, which is more likely to contribute to the reduction in salt intakes [45]. Research currently underway to look at the potential use of salt substitutes in China [46] is likely to provide a more viable approach to salt reduction in China, as well as being more effective in countries where the main source of sodium in the diet is salt added during cooking and at the table including Vietnam, Thailand and India.

The use of education campaigns is a key element of almost every salt reduction strategy and common to all strategies reporting a reduction in salt intake to date. Whilst the quality of the before and after surveys used in the seven countries reporting improved consumer KAB is not clear and likely to be subject to self-report bias, if the surveys are consistent, it can be a useful measure for change [39].

### Strengths and Limitations

This is the first comprehensive review of countries’ progress towards achieving the new global target for salt reduction. Questionnaires were sent to countries identified as having salt reduction initiatives and/or a country salt reduction contact person through a literature search and regional WHO representatives and experts. Although not all country contacts could be identified and there were some non-respondents, it is unlikely any major salt reduction initiatives were missed. The comprehensive database of national salt reduction initiatives and country contacts established as a result of this review will enable us to regularly update the information in future.

Much of the information was obtained from country questionnaires which relied on the knowledge and potentially subjective opinion of one country representative, usually from a government agency. Other stakeholders that often play an important role in salt reduction efforts, such as NGOs and industry organisations, were not requested to complete the questionnaire. Subsequent reviews of this kind might consider consulting with a wider range of stakeholders. However, in many cases, the questionnaire respondent acknowledged consulting other relevant people therefore initiatives are not likely to have been overlooked.

Similarly, whilst it is recognised that sub-national governments are implementing salt reduction strategies in their local jurisdiction, particularly healthy food procurement policies in public institution settings, this was not included as the review focused on national strategies. A review of sub-national salt reduction efforts and their impact would be useful to understand their contribution.

A key strength of this review is the systematic and comprehensive method of data collection. Although some salt reduction activities may have been missed in the previous review as it was not as comprehensive, an assessment of available start dates shows this did not affect the comparison of salt reduction strategies over time. The questionnaire was based on pre-defined categories from a previous review but expanded to encompass new elements of salt reduction strategies reflecting advancements on this issue. Future reviews should also include questions about national strategies to reduce iodine deficiency in view of the need to ensure that the two programs are effectively co-ordinated.

Whilst a key strength of the review is the fact that it encompasses a comprehensive search of the grey literature including government reports, presentations or questionnaires completed by country program officers, a parallel limitation of this is that the methodological rigor behind some of the reports is unknown. In particular, the robustness of the studies used as the basis for reported reductions in salt intake, salt levels in foods or changes in consumer KAB were not assessed and therefore should be interpreted with caution. The primary objective of this study was to provide a comprehensive overview of reported activity. A parallel Cochrane review of national salt reduction interventions currently underway will focus on the impact of the interventions [24]. The two studies taken together will add considerably to the body of evidence on the effectiveness and characteristics of national salt reduction programs.

## Conclusion

Population salt reduction is considered as a ‘best buy’ intervention for the prevention of NCDs [1] and this review exemplifies that countries of all regions and income levels are able to implement such programs. The significant increase in number of national salt reduction strategies and countries reporting an impact in relation to one or more outcome measures represents some progress towards the global salt target. However the scope of some existing salt reduction initiatives need expanding and more robust monitoring is required to ensure strategies are having optimal impact. Rigorous evaluation will also explain which elements are important to the success of programs as currently the implementation of initiatives is varied.

Our results also highlight less than half of the programs are implemented in LMICs. Urgent action is required to address this given that 80% of NCD deaths occur in LMICs [1]. Furthermore, it is projected that the greatest increases in NCDs will be in the WHO regions of Africa, South-East Asia and the Eastern Mediterranean [1]—the three regions currently with the least salt reduction activity. Increased support to implement salt reduction strategies in LMICs and robust evaluations of ongoing programs is imperative to ensure the targeted 30% reduction in mean population salt intake is achieved by 2025, which in turn will prevent millions of deaths worldwide.

## Supporting Information

S1 PRISMA Checklist PRISMA Checklist. (DOC)

S1 Annex Peer-reviewed Literature Search Strategy in MEDLINE. (DOCX)

S2 Annex Questionnaire sent to country salt reduction program leaders. (DOCX)

S3 Annex Characteristics of salt reduction strategies extracted. (DOCX)
