# Supplementary material for: Salt Reduction Initiatives around the World – A Systematic Review of Progress towards the Global Target
Source: PLoS One. 2015 Jul 22;10(7):e0130247. doi: 10.1371/journal.pone.0130247 (PMC4511674; doi:10.1371/journal.pone.0130247)
Supplement: S1 Annex — (DOCX) [file pone.0130247.s002.docx]

**Annex 1** Peer-reviewed Literature Search Strategy in MEDLINE

1. sodium, dietary/ or sodium chloride, dietary/
2. Sodium Chloride/
3. Sodium Glutamate/
4. sodium benzoate/ or sodium nitrite/
5. Sodium Hydroxide/
6. Diet, Sodium-Restricted/
7. (monosodium glutamate* or MSG or sodium additive* or sodium alginate* or sodium benzoate* or sodium caseinate* or sodium citrate* or sodium chloride* or sodium glutamate* or sodium hydroxide* or sodium nitrate* or sodium nitrite* or sodium phosphate* or sodium propionate* or sodium saccharin* or sodium sulfite*).tw.
8. ((salt or sodium) adj10 (reduc* or target* or cutback* or decreas* or limit* or consumption)).tw.
9. ((diet* or nutrition* or food or intake) adj10 (salt or sodium)).tw.
10. 1 or 2 or 3 or 4 or 5 or 6 or 7 or 8 or 9
11. Food, Formulated/
12. Food-Processing Industry/
13. food technology/ or food analysis/ or food preservation/
14. Food Industry/
15. 12 or 13 or 14
16. (adjust* or alter* or change or changing or control* or decreas* or limit* modify or modified or new or reduce or reducing or reduction* or reformulat* or redevelop* or restrict*).tw.
17. 15 and 16
18. ((adjust* or alter* or change or changing or control* or decreas* or limit* or modify or modified or new or reduce or reducing or reduction* or reformulat* or redevelop* or restrict*) adj10 (recipe* or food or foods or formula* or ingredient*)).tw.
19. 11 or 17 or 18
20. taxes/ or tax exemption/
21. Government Programs/
22. financing, organized/ or financing, government/
23. "Cost Sharing"/
24. (pricing or cost or costs or subsidi*).tw.
25. (taxation or taxes or subsid*).tw.
26. (financial adj3 (incentive* or disincentive*)).tw.
27. 20 or 21 or 22 or 23 or 24 or 25 or 26
28. Nutrition Policy/
29. exp Food Service, Hospital/
30. Food Services/
31. schools/ or schools, nursery/
32. Workplace/
33. Prisons/
34. Universities/
35. Child Day Care Centers/
36. ((food* or menu or nutrition*) adj5 (buy* or procur* or purchas* or stock*) adj5 (guideline* or policy or policies or practice* or standard*)).tw.

37. ((cafeteria* or diet or food* or menu* or nutrition*) adj10 (childcare or child-care or college* or daycare* or day-care* or fitness centre* or fitness center* or hospital* or leisure center* or leisure centre* or preschool* or pre-school* or prison* or public facilit* or recreation center* or recreation centre* or recreation facilit* or school* or universit*)).tw.
38. 28 or 29 or 30 or 31 or 32 or 33 or 34 or 35 or 36 or 37
39. Advertising as Topic/
40. ((market* or adverti* or promot*) adj10 (adolescent* or adolescence or child or children or teenager* or teens or young people or youth*)).tw.
41. 39 or 40
42. Food Labeling/
43. Food Packaging/lj, st [Legislation & Jurisprudence, Standards]
44. ((food* or nutrition* or diet*) adj10 (facts or information or label* or symbol* or warning*)).tw.
45. health check.tw.
46. 42 or 43 or 44 or 45
47. nutrition surveys/ or diet surveys/
48. communications media/ or exp mass media/
49. Social Marketing/
50. health education/ or exp consumer health information/ or health fairs/
51. exp Health Promotion/
52. Information Dissemination/
53. newspapers/ or periodicals as topic/
54. computer communication networks/ or internet/ or blogging/ or social media/
55. Electronic Mail/
56. ((communicat* adj2 campaign*) or (information adj2 campaign*) or mass media or newspaper* or television* or radio* or (public adj2 campaign*) or (national adj2 campaign*) or public information).tw.
57. (blog* or email* or facebook or internet or magazine* or mobile device* or PDA or SMS or smartphone* or social media or text messag* or twitter or web).tw.
58. (health education or health information or health promotion).tw.
59. 47 or 48 or 49 or 50 or 51 or 52 or 53 or 54 or 55 or 56 or 57 or 58
60. 19 or 27 or 38 or 41 or 46 or 59
61. 10 and 60
62. exp animals/ not humans.sh.
63. 61 not 62
