# Supplementary material for: Salt Reduction Initiatives around the World – A Systematic Review of Progress towards the Global Target
Source: PLoS One. 2015 Jul 22;10(7):e0130247. doi: 10.1371/journal.pone.0130247 (PMC4511674; doi:10.1371/journal.pone.0130247)
Supplement: S2 Annex — (DOCX) [file pone.0130247.s003.docx]

**Annex 2** Questionnaires sent to country salt reduction program leaders

**POPULATION SALT REDUCTION COUNTRY QUESTIONNAIRE**

**Name of country:**

|  |
| --- |

**YOUR NAME, ORGANISATION and POSITION:**

|  |
| --- |

**YOUR CONTACT DETAILS:**

|  |
| --- |

**Please answer the following questions as accurately as possible or if you prefer, please attach the relevant documents and web links that provides answers to the questions.**

**1. National salt reduction initiatives**

**1. Is there or has there previously been any program, policy or initiative designed to reduce population levels of salt/sodium intake in your country?** *This includes initiatives led by government, industry, non-governmental organizations (NGOs) or any other agency, and may include any of the following: regulation, product reformulation, labelling, consumer awareness/ education, dietary target development, strategy development, monitoring/surveillance, research etc.*

| **YES** *(cont. ques 1)* | **NO** *(go to ques 2)* | **PLANNED** *(cont. ques 1)* |
| --- | --- | --- |

| **Name of initiative:** | | | **Timescale:** |
| --- | --- | --- | --- |
| **Is there a national target for dietary salt intake?** | YES  NO  PLANNED | If YES, what is the target? ….…... | |
| **Who is the lead agency of the national salt reduction initiative?** | Government  NGO  Food Industry  Other, please specify: ….…... | | |
| **Is the initiative part of a broader program?** | YES  NO, the initiative is a salt specific program | | |
| **Are there any NGOs or advocacy organisations active on salt in the country?** | YES  NO | | |

**2. Population salt intake**

**Has any work been done to measure levels of salt/sodium intake in your country?**

| **YES** *(cont. ques 2)* | **NO** *(go to ques 3)* | **PLANNED** *(cont. ques 2)* |
| --- | --- | --- |

|  | **Year** | **Method** (E.g. dietary survey, 24 hour urine collection, spot urine.) | **Salt intake** (Total average, male average & female average) |
| --- | --- | --- | --- |
| **Please provide details on all the measurements of population salt intake in the country** (Insert additional rows if needed) |  |  |  |
|  |  |  |  |
|  |  |  |  |
|  |  |  |  |

**Has any work been done to measure the addition of salt or salty condiments to home cooking and/or at the table?**

| **YES. If yes, please specify how is it measured** ….…... | **NO** | **PLANNED** |
| --- | --- | --- |

**3. Salt levels in foods or meals**

**Has any work been done to determine the salt/sodium levels in foods or meals?**

| **YES** *(cont. ques 3)* | **NO** *(go to ques 4)* | **PLANNED** *(cont. ques 3)* |
| --- | --- | --- |

|  | **Year** | **Method** (E.g. food analysis, survey, database, industry self-report) | **Food categories collected** | **Reduction in salt/sodium content demonstrated** |
| --- | --- | --- | --- | --- |
| **Please provide details on all the measurements of salt levels in foods or food categories in the country.** (Insert additional rows if needed) |  |  |  | YES. Please specify which foods: ….…...  NO |
|  |  |  |  | YES. Please specify which foods: ….…...     NO |

**4. Consumer knowledge, attitudes and behaviour**

**Has any work been done to determine consumer knowledge, attitude and behaviour (KAB) in relation to salt/sodium?**

| **YES** *(cont. ques 4)* | **NO** *(go to ques 5)* | **PLANNED** *(cont. ques 4)* |
| --- | --- | --- |

|  | **Year** | **Method** (focus group, survey) | **Improvements in consumer KAB demonstrated** |
| --- | --- | --- | --- |
| **Please provide details on all the measurements of consumer knowledge, attitudes and behaviours in relation to salt in the country** (Insert additional rows if needed) |  | Survey  Focus group  Other | YES  NO |
|  |  | Survey  Focus group  Other | YES  NO |

**5. Implementation strategies**

**5A. ENGAGEMENT WITH INDUSTRY & REFORMULATION
Does your strategy include work with industry to achieve salt/sodium reduction in foods?**

| **YES** *(cont. ques 5a)* | **NO** *(go to ques 5b)* | **PLANNED** *(cont. ques 5a)* |
| --- | --- | --- |

| **Name of initiative & year implemented** |  | | |
| --- | --- | --- | --- |
| **Agency/organization taking the lead to engage industry** | Government  NGO | Industry  Other, please specify: ….…... | |
| **Is the approach voluntary or mandatory?** | Voluntary  Mandatory for all food categories (eg maximum salt content in food)  Mandatory for certain food categories such as ……………. | | |
| **Approach to work with industry** | Meetings with companies  Voluntary commitments to salt reduction from companies  Cross-sectoral agreements to salt reduction (e.g. all bread manufacturers)  Targets for salt levels in foods  Taxation for high salt products  Other, please specify ……………. | | |
| **If salt reduction targets have been used, which food categories have reformulation targets:** | Breads  Processed meats  Convenience/Ready meals  Breakfast cereals  Cheeses | | Butter and margarines  Salty snacks  Biscuits and cakes  Soups and Sauces,  Other, please specify ……………. |

**5B. CONSUMER EDUCATION/ BEHAVIOUR CHANGE
Does your strategy include activities to raise awareness/change behaviour on salt/sodium?**

| **YES** *(cont. ques 5b)* | **NO** *(go to ques 5c)* | **PLANNED** *(cont. ques 5b)* |
| --- | --- | --- |

| **Name of initiative & year implemented** |  |  |
| --- | --- | --- |
| **Agency / organization taking the lead** | Government  NGO  Industry  Other, please specify: | Government  NGO  Industry  Other, please specify: |
| **Approach** | Social marketing (e.g. campaigns)  TV advertising  Events  Other, please specify: | Social marketing (e.g. campaigns)  TV advertising  Events  Other, please specify: |

**5C. FRONT OF PACK LABELLING
Has your country introduced front of pack labelling to indicate nutritional or salt/sodium content of food?**

| **YES** *(cont. ques 5c)* | **NO** *(go to ques 5d)* | **PLANNED** *(cont. ques 5c)* |
| --- | --- | --- |

| **Name of initiative & year implemented** |  |
| --- | --- |
| **Agency / organization taking the lead** | Government  Industry  NGO  Other |
| **Is the approach voluntary or mandatory?** | Voluntary  Mandatory for all food categories  Mandatory for certain food categories such as ……………. |
| **Approach/detail of the label** (traffic light code, logo, % daily intake) | Traffic light code  Other logo or symbol  Warning labels  Percentage of daily intake  Other, please specify ……………. |

| **YES** *(cont. ques 5d)* | **NO** *(go to ques 6)* | **PLANNED** *(cont. ques 5d)* |
| --- | --- | --- |

**5D. WORK IN SPECIFIC SETTINGS
Does the strategy include work on salt/sodium in particular settings such as schools, hospitals or workplaces?**

| **Name of initiative & year implemented** |  | |
| --- | --- | --- |
| **Setting** | School  Hospital | The workplace  Other, please specify ……………. |
| **Approach to work in particular settings** | Education  Procurement policy | Voluntary Guidelines  Other, please specify ……………. |

**6. Evaluation**

**Has your country done any work to evaluate the overall effectiveness of salt/sodium reduction strategy, in addition to monitoring salt intake, salt levels and consumer KAB? (For example process evaluation or cost effectiveness analysis)**

| **YES** *(cont. ques 6)* | **NO** *(end of survey)* | **PLANNED** *(cont. ques 6)* |
| --- | --- | --- |

**For each evaluation, please specify:**

(please copy and paste the table below as needed, one table per evaluation)

| **Year evaluated** |  |
| --- | --- |
| **Evaluation approach** |  |
| **Results  (Has it had an impact?)** |  |
| **Has a cost-effective analysis been undertaken?** | YES  NO  PLANNED |
| **Was the program cost-effective?** | YES  NO |

**Please attach any documents and website links relevant to the questions.**

**THANK YOU FOR COMPLETING THIS QUESTIONNAIRE. We value your time. Please send the completed questionnaire and relevant documents to Kathy Trieu on** [**ktrieu@georgeinstitute.org.au**](mailto:ktrieu@georgeinstitute.org.au)
