# Supplementary material for: Salt Reduction Initiatives around the World – A Systematic Review of Progress towards the Global Target
Source: PLoS One. 2015 Jul 22;10(7):e0130247. doi: 10.1371/journal.pone.0130247 (PMC4511674; doi:10.1371/journal.pone.0130247)
Supplement: S3 Annex — (DOCX) [file pone.0130247.s004.docx]

**Annex 3** Characteristics of salt reduction strategies extracted

**Leadership and strategic approach**

1. Leadership – the organization leading or coordinating the programme
2. Dietary targets – presence of an agreed national population target for salt consumption with target amount.
3. Programme specificity – whether the strategy was salt-specific or a part of a broader health programme.
4. NGO/advocacy action – the presence of consumer/advocacy organizations working on salt

**Baseline assessments and monitoring**

1. Salt intakes – estimated mean baseline salt intakes and method of measurement
2. Salt levels in foods – whether countries had recorded salt levels in processed foods and method of measurement
3. Consumer awareness – whether countries had a baseline measure of consumer awareness or behaviours
4. Monitoring – whether or not monitoring systems are in place in relation to each of these criteria.

**Implementation strategies**

1. Food reformulation – whether the programme included work with the food industry to reformulate foods and whether the approach was voluntary or mandatory
2. Targets for salt levels in foods **-** whether the program of work with industry included the establishment of voluntary or mandatory salt level targets for foods
3. Consumer behaviour – whether there was a consumer awareness campaign and whether this was led by a NGO or Government.
4. Labelling – whether any new front-of-pack labelling scheme such as warnings, traffic lights, percentage daily intake or Guideline Daily Amount, or symbol or logo scheme had been introduced as part of the salt reduction programme and whether this was voluntary or mandatory.
5. Taxation on high salt products **–** whether the country had established a tax on high salt products
6. Work in public institution settings – whether there were any salt reduction strategies targeting public institution settings including schools, hospitals, workplaces or other public institutions through education about salt and health, food procurement policy with a sodium/salt criteria, voluntary guidelines for salt/sodium levels in foods or other activities

**Evaluation**

- 1. Change in salt intake- whether the country reported changes in population salt intake, what was the assessment method and what were the baseline and follow-up salt intake
  2. Change in salt levels in foods- whether the country had reported changes in salt levels of foods, what was the methods of measurement and what the baseline and follow-up salt levels were in specific food categories.
  3. Change in consumer knowledge, attitudes and behaviour (KAB)- whether the country reported changes in consumer knowledge, attitudes and behaviour in relation to salt, what was the methods of measurement and what were the baseline and follow-up data
